# Partial Agonism of Taurine at Gamma-Containing Native and Recombinant GABA_A_ Receptors

**DOI:** 10.1371/journal.pone.0061733

**Published:** 2013-04-30

**Authors:** Olaf Kletke, Guenter Gisselmann, Andrea May, Hanns Hatt, Olga A. Sergeeva

**Affiliations:** 1 Department of Cell Physiology of the Ruhr-University, Bochum, Germany; 2 Department of Neurophysiology, Medical Faculty of Heinrich-Heine University, Düsseldorf, Germany; INSERM U901, France

## Abstract

Taurine is a semi-essential sulfonic acid found at high concentrations in plasma and mammalian tissues which regulates osmolarity, ion channel activity and glucose homeostasis. The structural requirements of GABA_A_-receptors (GABA_A_R) gated by taurine are not yet known. We determined taurine potency and efficacy relative to GABA at different types of recombinant GABA_A_R occurring in central histaminergic neurons of the mouse hypothalamic tuberomamillary nucleus (TMN) which controls arousal. At binary α_1/2_β_1/3_ receptors taurine was as efficient as GABA, whereas incorporation of the γ_1/2_ subunit reduced taurine efficacy to 60–90% of GABA. The mutation γ_2F77I_, which abolishes zolpidem potentiation, significantly reduced taurine efficacy at recombinant and native receptors compared to the wild type controls. As taurine was a full- or super- agonist at recombinant α_x_β_1_δ-GABA_A_R, we generated a chimeric γ_2_ subunit carrying the δ subunit motif around F77 (MTVFLH). At α_1/2_β_1_γ_2(MTVFLH)_ receptors taurine became a super-agonist, similar to δ-containing ternary receptors, but remained a partial agonist at β_3_-containing receptors. In conclusion, using site-directed mutagenesis we found structural determinants of taurine’s partial agonism at γ-containing GABA_A_ receptors. Our study sheds new light on the β_1_ subunit conferring the widest range of taurine-efficacies modifying GABA_A_R function under (patho)physiological conditions.

## Introduction

Taurine (2-aminoethane sulfonic acid) is very abundant in plasma and mammalian tissues including brain, where it regulates osmolarity, ion channel activity, neuronal growth and metabolism [1]–[4]. It remains controversial whether taurine can be called “neurotransmitter”: some but not other studies reported accumulation of taurine in the synaptic vesicles [5]; [6] and action potential-dependent release [7]. Taurine concentrations range from 3 to 9 mM in different species and brain regions and may reach 20 mM or higher in intracellular compartments [8]. Intracellular concentrations of taurine in the brain are about 400 times higher then extracellular [9] due to the high-affinity uptake system [10]. Taurine release from different CNS cells is observed under pathophysiological conditions such as hypoosmotic stress, ischemia or acute hyperammonemia, where its interaction with the receptors for inhibitory neurotransmitters GABA and glycine plays a neuroprotective role [8]. In many brain areas taurine in concentrations below 1 mM activates glycine but not GABA_A_ receptors, except for the ventrobasal thalamus, where it activates α_4_β_2_δ GABA_A_R-type at physiological concentrations (10–100 µM) [11]. Mice deficient in taurine show impaired GABAergic inhibition in the striatum [12], indicating yet unrecognised role of taurine for the proper GABAergic signalling. If molecular structure of taurine binding site at different glycine receptor types are known [13]; [14], taurine binding site at GABA_A_ receptor was not yet systematically analysed. Efficacy and potency of taurine is so far only known for a few subunit combinations. Taurine acts as a full agonist at α_1_β_3_ and a partial agonist at α_1_β_3_γ_2_ receptors [15]; at α_4_β_2_δ receptors taurine elicits even greater currents than GABA [11] and at α_6_β_2_δ GABA_A_R taurine is a partial agonist, with variable EC_50_s depending on the expression level [16]. The molecular mechanism that determines the efficacy of taurine at GABA_A_Rs is unknown. A comparative analysis of taurine gating of GABA_A_R containing different β subunits was not yet performed. GABA_A_Rs are heteropentameres composed of five subunits. A multitude of subunits can assemble to functional receptors (α_1–6_, β_1–3_, γ_1–3_, δ-, ε-, θ-, π-and ρ_1–3_) [17]. According to the current view GABA_A_Rs are composed of two α, two β and one γ (or δ) subunits aligned γ-β-α-β-α counter-clockwise when viewed from the synaptic cleft [18]. Mutational analysis studies demonstrated that the agonist binding site is located at the α/β interface and the benzodiazepine binding site at the α/γ interface [17]; [19]; [20]. The binding sites for the partial agonists at GABA_A_R are unclear [21]. The largest population of GABA_A_Rs in the rat brain has a subunit composition of α_1_β_2_γ_2_, whereas α_2_β_3_γ_2_ and α_3_βγ_2/3_ together constitute the next most prevalent subtypes [22]; [23]. Several subunit combinations such as α_5_β_3_γ_2_ [22] and α_4/6_β_2/3_δ [23]; [24] are found exclusively extrasynaptically, with the former type expressed in the hypothalamus. Histaminergic neurons from the tuberomamillary nucleus (TMN) of the hypothalamus were selected for the present study as functional and structural features of their GABA_A_ receptors were previously characterised with the α1, α2, α5, β1, β3, γ1, γ2, ε, but not δ subunit- transcripts being regularly detected [25]–[28]. We compare now the taurine-sensitivity of native GABA_A_R versus selected GABA_A_R compositions recombinantly expressed in *Xenopus* oocytes. As we aimed to compare properties of recombinant GABA_A_R with the native receptors expressed in hypothalamic neurons we restricted the number of investigated subunits and receptor types to those present in TMN neurons [27]; [28]. We report that incorporation of the γ subunit reduces taurine efficacy. With the help of site-directed mutagenesis we describe structural determinants for the partial agonism of taurine at γ-containing GABA_A_Rs.

## Materials and Methods

### Electrophysiology in Native Neurons

Experiments were conducted according to the Animal Protection Law of the Federal Republic of Germany (Tierschutzgesetz BGBI.I,S.1206, revision 2006) and European Communities Council directive regarding care and use of animals for experimental procedures (86/609/EEC). Approval by the Ethics Committee for this kind of experiment is not necessary in accordance with the Animal Protection Law of the Federal Republic of Germany (§ 8 Abs.1 Tierschutzgesetz). All efforts were made to minimize the number of animals and their suffering. Brain tissue was removed from mice after decapitation by appropriately trained staff with approval of LANUV NRW (Landesamt für Umwelt, Natur und Verbraucherschutz Nordrhein Westfalen, Düsseldorf), permission number 058/91.

Five to eight week old male mice carrying a point mutation on GABA_A_R γ_2_ subunit (γ_2F77I_) further referred as KI (knock-in) mice and their wild type littermates were generated and genotyped as described previously [29]. Slice preparation, isolation of histaminergic neurons with the help of papain, whole-cell patch-clamp recordings in voltage clamp mode, fast drug application and single cell RT-PCR procedures was done as previously described [26]; [27]. Briefly, sterile patch electrodes were filled with the following solution: 140 mM KCl, 2 mM MgCl_2_, 0.5 mM CaCl_2_, 5 mM EGTA, and 10 mM HEPES/KOH (pH 7.2). After establishment of the whole-cell configuration (Vh = −50 mV), an acutely isolated cell was lifted into the major chute of the application system, where it was continuously perfused with the sterile control bath solution. The substances were applied through a glass capillary 0.08 mm in diameter. All solutions flowed continuously, gravity-driven, at the same speed and lateral movements of the capillaries exposed a cell either to control- or test-solutions. The kinetics of solution exchange at the open electrode tip were characterized by an exponential rise time constant of 7 ms, whereas the maximal GABA-evoked responses reached their maximum up to 2 times slower; thus peak responses represented the sum of activation, desensitization and delay of solution exchange around the large (15–25 µm) neurons (see Schubring et al [30]). For the comparison of apparent desensitization kinetics between zinc sensitive and zinc resistant neurons, only cells with a rise time constant below 10 ms were considered. Experiments were conducted and analyzed with commercially available software (TIDA for Windows, HEKA, Lambrecht, Germany). Fitting of concentration - response data points was done as previously described [25]; [26]. Post-hoc identification of recorded TMN neurons and GABA_A_R analysis was done with single cell RT-PCR according to the previously published protocols [27]; [28]. Real-time RT - PCR was used for the semiquantitative analysis of γ_2_ subunit expression in TMN (relative to the β -actin endogenous control according to the “2^−ΔΔCt^ “(ΔFold) method as in [27]. After the final cycle the PCR were subjected to a heat dissociation protocol (PE Biosystems 5700 software). Each PCR product showed a single peak in the denaturation curve. Standard curves were obtained with the sequential dilution of one cDNA sample (from KI mouse #1). From these curves the linear regression coefficient (r = −0.99) and efficiency (E = 1.8) for the β –actin and γ_2_ subunit – cDNA amplification (r = −0.98, E = 1.9) were calculated, where E = 10^[−1/slope]^. Expression levels of the γ_2_-subunit in each sample are normalized to the sample with minimal expression (for this sample: ΔΔC = 0, 2^−ΔΔCt = ^1). The following primers were used: up: 5′-tat gtD aac agc att ggW ccW gt -3′ and lo: 5′-acc atc att cca aat tct cag cat-3′. The size of the PCR product (234 b.p.) was verified by electrophoresis in 2% agarose gel, whereas its identity with the known mouse γ_2_-subunit cDNA (M86572, Genbank) was confirmed by sequencing.

### Expression of Recombinant GABA_A_ Receptors and Electrophysiology in Xenopus Oocytes

GABA_A_R subunit cDNAs were obtained as follows: rat α_1_ and β_1_ cDNAs were prepared using standard molecular biology procedures. Mouse γ_2L_, α_2_, and human β_3_ and δ cDNA were obtained from RZPD (Berlin, Germany). Chimeric γ_2(δ 74–79)_ was generated using overlap extension PCR [31] with the following primer pairs for the exchanged area: fw-γ_2(δ 74–79)_ ‘5-atg gaa tat aca atg acg gtg ttc ctg cac cag agc tgg cgg gac aga cgt ttg aaa ttt aac-3′ and rev- γ_2(δ 74–79)_ ‘5-gtt aaa ttt caa acg tct gtc ccg cca gct ctg gtg cag gaa cac cgt cat tgt ata ttc cat-3′. All cDNAs were subcloned into pSGEM (courtesy of M. Hollmann, Bochum, Germany). Plasmids were linearized with PacI restriction endonuclease and corresponding cRNA was synthesized using the AmpliCap T7 high-yield message marker kit (Epicentre, Madison, WI), following the manufacturers protocol. 5 to 15 ng of the mixture of cRNAs with a ratio of 10∶1:10 for αβγ/δ was injected into every oocyte to prevent subpopulations of β homomultimeric GABA_A_R [32]. Two to six days after injection of cRNA, oocytes were screened for receptor expression by two-electrode voltage-clamp recording. Electrodes were made using a Kopf vertical micropipette puller and filled with 3 M potassium chloride, giving resistances of 0.1–0.5 MΩ. Eggs were placed in an oocyte chamber and superfused with Frog-Ringer’s solution (115 mM NaCl, 2.5 mM KCl, 1.8 mM CaCl_2_, 10 mM Hepes, pH 7.2). Current signals were recorded with a two-electrode voltage - clamp amplifier (TURBO TEC-03, npi, Tamm, Germany) and pCLAMP software (Axon Instruments, Union City, CA) or CellWorks (npi, Tamm, Germany) depending on the setup used. The membrane potential was clamped at −40 to −60 mV. All experiments were performed at room temperature. Complete concentration - response curves for GABA and taurine were recorded on the same oocyte. These agonists of GABA_A_R were dissolved in Frog-Ringer and applied in a volume of 200 µl into the entrance tube of the recording chamber, totally exchanging the bath solution within a second. Currents were analyzed using pCLAMP 10 software. Dataset was processed in Excel (Microsoft Corporation, Redmond, WA). Curve fitting by the 3 parameter Hill equation and statistics (t - test) was done using SigmaPlot V8.0 (Systat Software, San Jose, CA). Taurine efficacy was determined by the maximum of the taurine concentration - response curve calculated by the 3 parameter Hill equation in relation to the maximum current of the GABA concentration - response curve. Proper γ subunit integration into the GABA_A_R was analysed using zinc. Ternary αβγ GABA_A_Rs are insensitive to low micromolar concentrations of zinc, whereas binary αβ receptors are inhibited by those concentrations [33] (Figure S1). Delta (δ) - containing GABA_A_R have intermediate zinc sensitivities [34], therefore our criterion for δ subunit integration was the modulation of GABA - evoked currents by tracazolate (Figure S2). In accordance with Thompson et al. [35] we found that tracazolate potentiates ternary δ - containing GABA_A_R to a larger extent than the corresponding binary α_x_β_x_ receptors.

### Drugs and Statistical Analysis

Gabazine (SR 95531) and tracazolate were obtained from Tocris-Biotrend (Köln, Germany). All other chemicals were obtained from Sigma-Aldrich (Taufkirchen, Germany). Drugs were diluted and stored as recommended. Statistical analysis was performed with the non - parametrical Mann - Whitney U-test if not indicated otherwise. Significance level was set at p<0.05. Data are presented as mean ± standard error of the mean (SEM).

## Results

### Taurine Efficacy and Potency at GABA_A_ Receptors Composed of α and β Subunits

In accordance with the study performed in HEK 293 cells by Dominguez-Perrot et al. [15] recombinant GABA_A_R composed of α_1_- and β_3_ -subunits in our study responded to GABA and to taurine with maximal currents of similar amplitude (Table 1). At receptors containing the β_1_-subunit taurine demonstrated super-agonism, eliciting maximal responses nearly two times larger than the maximal GABA responses (Table 1). When the α_1_-subunit was replaced by the α_2_-subunit GABA and taurine potencies were reduced at β_1_-containing receptors whereas taurine potency at β_3_-containing receptors was not affected. Taurine efficacy was independent of the α-subunit type and was determined by the β-subunit type.

**Table 1:** GABA- and taurine- gating of different GABA_A_ receptor types recombinantly expressed in Xenopus oocytes.

	GABA		Taurine			
	nHill	EC_50_ [µM]	Imax	nHill	EC_50_ [mM]	n
**α_1_β_1_**	**1.82±0.13**	**8.0±0.8**	**2.26±0.27**	**0.71±0.07**	**59.3±18.8**	4
α_1_β_1_δ	1.85±0.10	8.5±1.8	2.75±0.05*	0.60±0.02	162.6±33.4*	5
α_1_β_1_γ_2_	1.82±0.16	10.0±1.1	0.81±0.03*	1.20±0.04*	17.6±2.18	4
α_1_β_1_γ_2__(δ74–79)_	1.38±0.10*	13.2±1.6*	2.7±0.30*	0.48±0.02*	433.4±94.8*	7
α_1_β_1_γ_2F77I_	1.60±0.13	36.2±6.7*	0.53±0.10*	0.91±0.09	68.6±13.9	5
**α_1_β_3_**	**1.92±0.21**	**7.8±0.8**	**1.06±0.06**	**0.96±0.09**	**26.1±9.5**	5
α_1_β_3_δ	1.44±0.09*	21.4±8.1	0.74±0.04*	1.09±0.08	45.3±10.5	6
α_1_β_3_γ_2_	1.68±0.15	16.7±2.3*	0.63±0.09*	1.30±0.13*	86.3±16.5*	5
α_1_β_3_γ_2 (δ74–79)_	1.34±0.03*	48.4±14.2**	0.44±0.07*	0.94±0.09	92.9±16.3*	5
α_1_β_3_γ_2 F77I_	1.58±0.13	66.4±86.3*	∼0.21±0.04	n.p.	n.p.	5
**α_2_β_1_**	**1.86±0.19**	**18.0±2.1**	**1.80±0.14**	**1.12±0.16**	**81.8±24.9**	7
α_2_β_1_δ	1.72±0.11	19.1±1.4	1.94±0.11	1.02±0.09	67.8±15.9	6
α_2_β_1_γ_1_	1.35±0.10*	87.1±7.1*	0.64±0.03*	1.22±0.30	107.2±25.8	4
α_2_β_1_γ_2_	1.37±0.08*	71.2±12.5*	0.65±0.05**	1.06±0.06	120.3±26.0	5
α_2_β_1_γ_2 (δ74–79)_	1.88±0.04	15.1±0.4	1.43±0.06	0.98±0.07	40.5±6.9	7
α_2_β_1_γ_2 F77I_	1.68±0.06	58.2±3.9*	0.49±0.02**	1.29±0.02	76.1±3.9	5
**α_2_β_3_**	**1.48±0.21**	**12.9±6.5**	**1.03±0.04**	**1.07±0.08**	**24.5±11.2**	**4**
α_2_β_3_δ	1.47±0.06	13.5±1.0	1.10±0.03	1.29±0.03*	35.4±2.0	7
α_2_β_3_γ_1_	1.45±0.05	93.8±12.2*	∼0.44±0.04	n.p.	n.p.	4
α_2_β_3_γ_2_	2.12±0.01*	18.7±0.9	0.87±0.03*	1.14±0.11	51.2±19.8	4
α_2_β_3_γ_2__(δ74–79)_	1.47±0.05	42.8±5.8*	0.51±0.03*	1.11±0.07	137.1±26.2*	5
α_2_β_3_γ_2F77I_	1.65±0.13	68.8±7.4*	0.36±0.02*	1.29±0.12	174.4±28.7*	5

**Figure 1:**
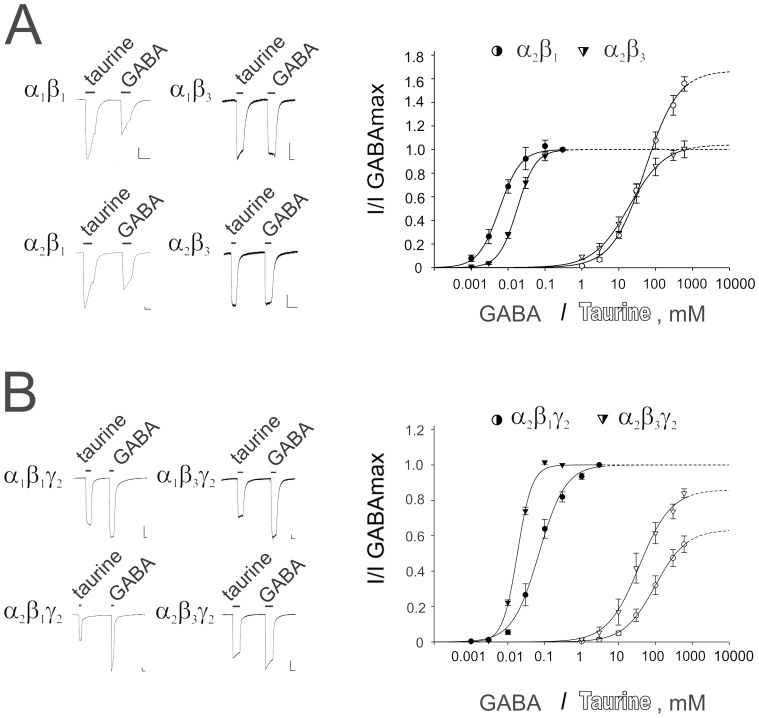
Comparison of taurine- and GABA-evoked maximal currents recorded from binary α_x_β_x_ (A) or ternary α_x_β_x_γ_2L_ (B) GABA_A_ receptors. Note that gating by taurine of γ_2_ subunit containing GABA_A_ receptors is significantly less efficacious compared to the corresponding binary receptors. Representative current traces (comparison of taurine (600 mM) - and GABA (0.1–1 mM) -evoked maximal currents at different receptor subtypes) are shown at the left. Scale markers represent 0.1 µA vertically and 20 s horizontally for all figures with oocyte recordings. Right: averaged concentration - response curves. Concentration of agonist (filled symbols for GABA -, open symbols for taurine - responses) is plotted versus normalized response amplitudes. Each individual measurement was first normalized to the observed maximal GABA - current amplitude and subsequently averaged. Number of investigated oocytes, Hill coefficients (nHill) and concentrations evoking a half - maximal response (EC_50_) are presented in Table 1.

GABA I _max_ = 1; Responses to different taurine concentrations relative to I GABA max were fitted with a logistic Hill equation as shown in Fig. 1: EC_50_, nHill and maximal efficacy (relative to GABA), obtained from this curve-fit analysis are provided for each receptor type. Exceptions are marked with ∼ (in these two cases, the curve-fit failed due to the large deviation from mean of experimental values. Mean relative response amplitude (to 0.6 M taurine) is given). Values represent mean ± SEM. n.p. = not predictable. All values were compared to those seen in the corresponding binary (α_x_β_x_) receptor type (fat); significant difference is indicated (*p<0.05, **p<0.01, non - parametrical Mann - Whitney U-test).

### Presence of γ_2_-subunit Reduces Taurine Efficacy

When co-assembled with the α- and β-subunits the γ_2L_ subunit negatively affected taurine efficacy in all receptor types. At α_2_β_3_γ_2L_ taurine efficacy (0.87) showed the slightest but significant deviation in efficacy reduction compared to the binary α_2_β_3_ receptors (Table 1, Fig. 1). After integration of the γ_2L_ or γ_1_ subunit into β_1_-containing receptors, the efficacy of taurine was reduced to about 1/3 of the binary α_x_β_1_ receptor and taurine could be called a “partial agonist”. Previous studies have shown that the putative assembly signals, the residues determining selective co-assemblies of α-β or α-γ_2_, in GABA_A_Rs [36] as well as in nicotinic acetylcholine receptors [37], are adjacent to, or identical to the residues that actually form the ligand-binding site. Thus, the α_1_ residues 56–67, in particular glutamine 67 (α_1Q67_), are important for the assembly with the β_3_ subunit and are involved in the formation of the low affinity GABA-binding site [36]; [38]. The γ_2_A assembly signal (MEYTIDIFFAQTW) [36] which interacts with the α subunit includes phenylalanine at position 77 (F77). This residue plays an important role for the zolpidem modulation of γ_2_-containing GABA_A_R [39].

### Role of Zolpidem Binding Site for the GABA_A_R Gating by Taurine

**Figure 2:**
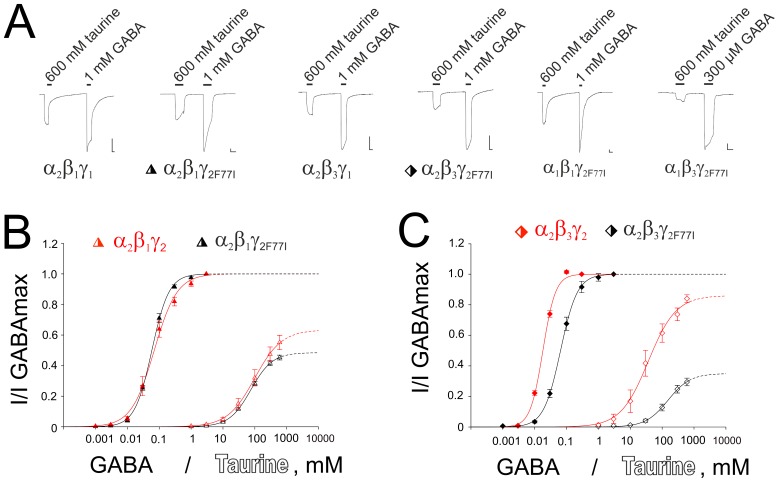
Mutation γ_2F77I_ reduces taurine efficacy at recombinant GABA_A_ receptors. (**A**) Representative current traces show responses to the maximal GABA and taurine concentrations at different receptor types. For two representative receptor types (marked with symbols) concentration-response plots for GABA (filled symbols) and taurine (open symbols) are shown in (**B**) and (**C**). Data obtained in corresponding wild type receptors (γ_2_ instead of γ_2F77I_) are plotted in red.

The mutation of phenylalanine to isoleucine at position 77 of GABA_A_R γ_2_ subunit (γ_2F77I_) leads to the loss of zolpidem-modulation of GABA-responses in recombinant and native receptors [29]; [39]. This is the only residue, which is different between the γ_2_ and the γ_1_ subunit within the putative assembly signal γ_2_A (see above). As taurine was significantly less efficient at α_2_β_3_γ_1_- than at α_2_β_3_γ_2_- receptors (Table 1) we generated a mutated γ_2F77I_ subunit using overlap extension PCR techniques [31]. Proper incorporation of γ_2F77I_ into GABA_A_R was verified by zinc-resistance (Figure S1). In all investigated receptor types taurine efficacy was significantly reduced at mutated compared to the corresponding wild type receptors: p<0.05 for α_1_β_1_γ_7F77I_; p<0.01 for α_2_β_1_γ_2F77I_; p<0.001 for α_2_β_3_γ_2F77I_ (Table 1, Fig. 2). As previous studies examining macroscopic (whole-cell currents) and microscopic (single-channel currents) kinetics of recombinant GABA_A_R with a mutation within the GABA-binding site came to the conclusion that the reduction in agonist potency (e.g. a 70-fold increase in EC_50_ for GABA after mutation β_2_-R207C) may be accompanied by the apparent reduction (by half) of the relative efficacy of a partial agonist (e.g. piperidine-4-sulfonic acid, P4S) under the slow, but not under the fast solution exchange conditions [40]. In order to control for this possibility we performed a correlation analysis between the relative potency of taurine (EC_50_ taurine/EC_50_ GABA) versus relative efficacy of taurine for all individual measurements from γ_7F77I_ –containing and corresponding WT receptors. There was no significant correlation (Pearson coefficient: −0.07, p = 0.75). Taurine was 1965±219 times (n = 15) and 1692±154 times (n = 11) less potent than GABA in KI and WT receptors, respectively (p = 0.8), but its relative efficacy was significantly lower in KI receptors (45.6±3.7% vs 74.6±3.7%, respectively, p = 0.0002).

### GABA_A_ Receptors in Zinc-resistant Neurons from Mutant γ_2F77I_ Mice show Reduced Taurine Gating

**Figure 3:**
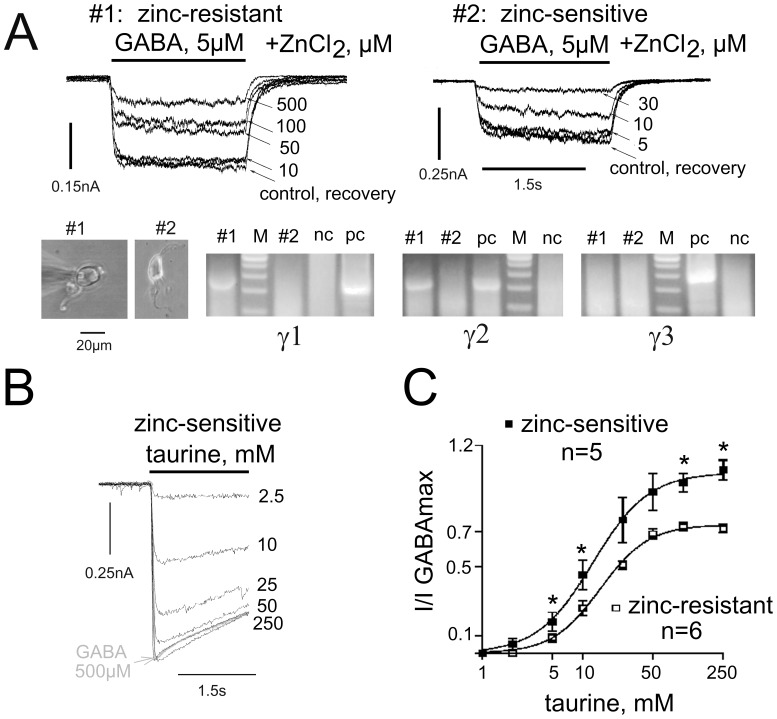
Zinc-sensitive TMN neurons show similar efficacies for GABA and taurine. (**A**) Zinc-inhibition of GABA-evoked currents in two representative neurons. Note that these neurons respond differently to ZnCl_2_ 10 µM. Block of the GABA-response by this concentration served as a criterion for the selection of “zinc-sensitive” neurons. (**B**) Photographs of two neurons and gels illustrating single-cell RT-PCR analysis of γ-subunit (γ1–γ3) expression. Note the lack of a detectable amount of γ-subunit transcripts in zinc-sensitive cell (#2). (**C**) Superimposed responses to different concentrations of taurine in comparison to the maximal GABA response recorded in one zinc-sensitive neuron. (**D**) Averaged concentration - response plots for the two neuronal groups. Significant difference between individual data points is indicated: * = p<0.05. The maximal taurine-evoked currents represented 100±5% (filled squares, EC_50_ = 12.6±0.6 mM, n = 5) vs 74±2% (open squares, EC_50_ = 14.9±0.9 mM, n = 6) of maximal GABA-evoked currents.

**Figure 4:**
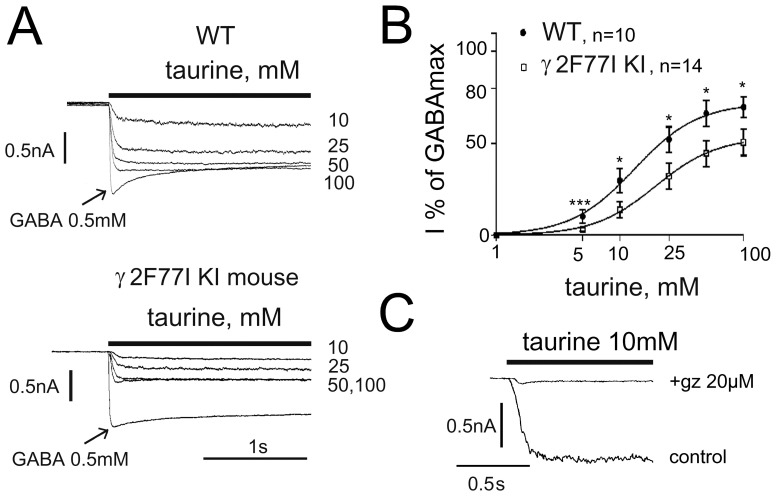
Gating of native GABA_A_ receptors by taurine is impaired by the mutation γ_2F77I_. (**A**) Whole-cell voltage-clamp recordings (V_h_ = −50 mV) from adult WT or KI mouse TMN neurons isolated from hypothalamic slices. Taurine evokes maximal responses (at 50 and 100 mM) which are comparable in amplitude to the maximal GABA (0.5 mM)-evoked currents in wild-type (WT) mouse but represents only half of the GABA-response in the knock-in (KI) γ_2F77I_ mouse. (**B**) Averaged concentration - response curves obtained from 10 WT and 14 KI neurons. Significant difference between individual data points is indicated: * p<0.05; *** p<0.005. (**C**) GABA_A_R- versus GlyR-involvement in taurine-responses was tested by the co-application of taurine with gabazine (gz, GABA_A_R antagonist). Amplitude of the remaining response was subtracted in each neuron from the control taurine response, to construct the concentration - response curves in (B).

Acutely isolated mouse TMN neurons responded to GABA with EC_50_s around 15 µM. There was no difference in GABA-sensitivity between γ_2F77I_ mice and their WT littermates. All data presented in the manuscript are obtained from neurons expressing histidine decarboxylase (cell identification with single-cell RT-PCR). In contrast to the rat [25], where GABA_A_Rs are “zinc-resistant” in all TMN neurons, about 30% of mouse TMN neurons are zinc-sensitive. No difference in the occurrence of zinc- sensitivity was found between γ_2F77I_ (knock-in, KI) mice and their WT littermates (27.7% and 27% of cells, respectively). GABA EC_15_ responses were inhibited by 30 µM of ZnCl_2_ in zinc-sensitive cells to 28.2±5.3% of control and by 10 µM to 52.1±5.9% of control (pooled data from 3 WT and 5 KI neurons, where a complete analysis of GABA_A_R expression with single-cell RT-PCR was successfully done, Fig. 3). In zinc-resistant cells, where 10 µM of zinc did not affect GABA-responses, inhibition by 30 µM zinc amounted to 74.1±2.2% of control (significantly different from “zinc-sensitive” cells; p<0.01). The apparent macroscopic desensitization of current responses to saturating GABA concentration (plateau/peak ratio at the end of a 2s-application period) amounted to 73.3±3.3% (n = 5) vs 67.6±3.7% (n = 10), in zinc-sensitive and zinc-resistant cells respectively (the difference is not significant). In 25% of the zinc-sensitive cells mRNAs encoding for γ subunits were not detected, whereas in the same cells α- and β-subunit transcripts were present. Two different γ subunits were never found coexpressed in zinc-sensitive neurons, whereas 48% of zinc-resistant cells coexpressed γ_1_ and γ_2_ subunits (p<0.05, Fisher’s test). All zinc-resistant cells (n = 21) expressed either γ_1_ (57%) or γ_2_ (90.5%) subunit or both. The detection frequency of any of the GABA_A_R subunits did not differ between 11 WT and 18 KI neurons (% of positive cells: WT vs KI): α_1_ in 18% vs 28%, α_2_ in 100% vs 94%, α_5_ in 18% vs 17%, β_1_ in 18% vs 44%, β_2_ in 9% vs 17%, β_3_ in 91% vs 78%, γ_1_ in 36% vs 50% and γ_2_ in 82% vs 83%. None of the cells expressed a detectable amount of γ_3_ subunit transcripts. Semiquantitative real-time PCR analysis of γ_2_ subunit expression revealed no difference in mRNA levels between TMN of γ_2F77I_ KI mice (n = 5) and their WT littermates (n = 5): 1.5±0.1 vs 1.5±0.2 (p = 0.83). In WT mice taurine was more effective (p<0.05) in “zinc-sensitive” cells compared to “zinc-resistant” ones (Fig. 3B and C). In “zinc resistant cells” taurine was significantly more efficient in WT (72±2.4% of maximal GABA-currents, n = 10) compared to KI mice (53±2%, n = 14; Fig. 4A and B). Neither taurine potency (13±1 mM vs. 19.4±1.3 mM) nor slope functions of dose-response curves (nHill 1.7±2 vs. 1.74±0.14) differed between WT and KI neurons. The GlyR-mediated component of taurine-responses was subtracted from each response-amplitude (remaining component after co-application of taurine and gabazine 20 µM, Fig. 4C).

### Super-agonistic Properties of Taurine at α_x_β_1_δ-receptors can be Transferred to the α_x_β_1_γ_2L_ Receptors by Introducing into the γ_2L_ Subunit the δ-motif: MTVFLH

**Figure 5:**
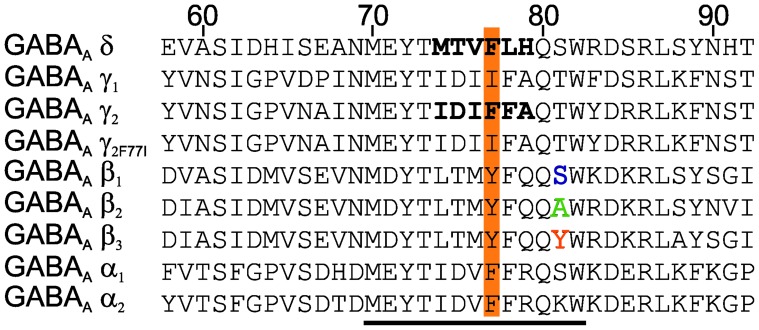
Sequence alignment of GABA_A_ receptor subunits between amino acids 58 and 92 (γ_2_ mouse numbering). Underlined is a putative assembly signal conserved in different GABA_A_ receptor subunits (36)). Note no difference between all three β- subunits in the putative assembly signal: MDYTLTMYFQQ_W with the exception for the position 81 (different residues are indicated in different colour). Interestingly, these coloured β subunit-specific residues were shown previously to affect stabilization of a homomeric assembly (45). Fat letters show δ: MTVFLH and γ_2_: IDIFFA motifs which were exchanged in the chimeric γ_2(δ74–79)_ subunit. Orange field indicates location of γ_2F77_ site involved in zolpidem binding as well as homologous or same residues at other GABA_A_R subunits.

**Figure 6:**
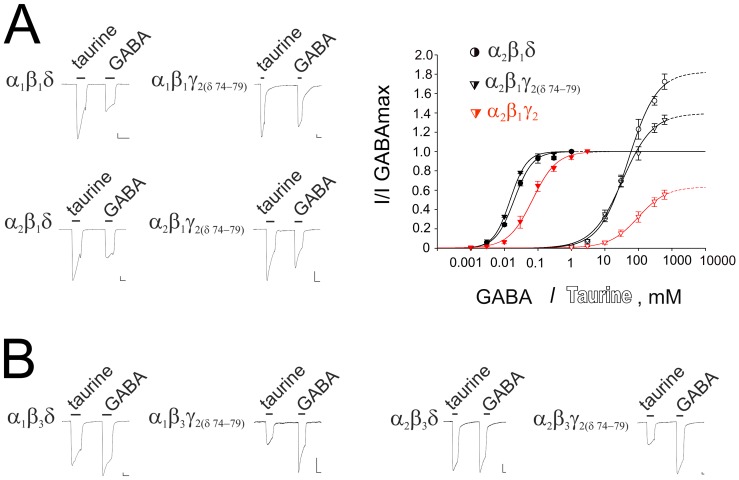
Chimeric α_x_β_1_γ_2(δ74–79)_ receptors show superagonistic properties of taurine. (**A and B)** Representative current traces (comparison of taurine (600 mM) - and GABA (0.3–3 mM) - evoked maximal currents) are shown for different receptor types. (**A**) Concentration - response curves for the β_1_ -containing receptors. Concentrations of agonist are plotted versus current amplitudes normalized on maximal GABA response (filled symbols for GABA -, open symbols for taurine - responses). Red curves are given for comparison with γ_2_ (WT) - containing receptors. Note the dramatic increase in taurine efficacy over GABA in chimeric β_1_ - containing GABA_A_R, which renders them similarity with the α_2_β_1_δ receptors.

This and previous studies show that δ-containing receptors are more potently and efficiently gated by taurine than γ-containing receptors. The molecular determinants for high sensitivity to taurine are unknown. We exchanged the γ_2_ motif around phenylalanine 77 which we found to be responsible for the reduced efficacy of taurine with the corresponding motif of the δ subunit (Fig. 5). The resulting chimeric receptors α_x_β_1_γ_2(δ74–79)_ displayed superagonistic properties of taurine, which did not differ significantly from the α_x_β_1_δ receptors (Table 1, Fig. 6). Interestingly, co-assembly of the chimeric γ_2_ subunit with α_x_ and β_3_ subunits did not render taurine agonism superior to GABA (Table 1). Chimeric α_2_β_3_γ_2(δ74–79)_ receptors were insensitive to zolpidem, like α_2_β_3_γ_2F77I_ or α_2_β_1_γ_1_ receptors (Figure S3). When GABA (at EC_10_) was co-applied with 1 µM zolpidem, the resulting currents represented 97±7% of control (n = 5). At wild type α_2_β_3_γ_2_ receptors the same concentration of zolpidem increased control GABA-response to 430±70% of control (n = 5). Thus, zolpidem insensitivity could be transferred from δ to γ_2_ through the motif MTVFLH.

## Discussion

We demonstrate that taurine gating depends on the type of β subunit and is negatively affected by the γ subunit of the GABA_A_R. The mutation γ_2F77I_ which makes the GABA_A_R zolpidem-insensitive reduces the efficacy of taurine-gating in recombinant and native GABA_A_ receptors. Substitution of the γ_2_ subunit motif around phenylalanine 77 (mouse γ_2_ subunit numbering) with the corresponding δ subunit motif (MTVFLH) results in a receptor with superagonistic properties of taurine in β_1_- but not in β_3_- containing receptors.

Our results obtained on recombinant GABA_A_R expressed in Xenopus oocytes are in line with a previous report on decreased efficacy and potency of taurine at ternary α_1_β_3_γ_2_ receptors compared to binary α_1_β_3_ receptors expressed in HEK293 cells [15]. This decrease in efficacy was accompanied by decreased taurine potency at α_1_β_3_γ_2_ and α_2_β_3_γ_2_ receptors (Table 1; present study and [15]). Interestingly, β_1_-coassembly with the γ_2_ subunit resulted in a reduction of taurine efficacy and potency in α_2_- but an increased potency in α_1_-containing pentamers in our study. We are aware of the technical limitations in our measurements of maximal efficacies and potencies of GABA_A_R agonists due to the slow speed of the solution exchange around an oocyte. Apparent efficacies and potencies were calculated from the peak responses which represent the sum of different processes such as fast kinetics of receptor activation and desensitization and the slow concentration ramp. Theoretical predictions formulated in a study by Wagner et al [40] are the following: i) the true maximal efficacy or open probability is underestimated in experiments on oocytes as desensitization during the agonist concentration ramp blunts the peak amplitude of the response; ii) the degree of this blunting depends on ligand affinity, such that high affinity ligands reach higher effective concentrations sooner during the agonist concentration ramp than do low affinity ligands. Although absolute efficacy and potency values can only be obtained from experiments recording single channel activity, our results from Xenopus oocytes are in line with those from HEK293 cells [15] and native neurons (present study) where a much faster solution exchange around smaller cells was achieved. Combining patch-clamp recordings from hypothalamic neurons with single-cell RT-PCR we observed the same structure-function relation for taurine gating of native GABA_A_R as seen in Xenopus oocytes. Thirty percent of mouse histaminergic neurons expressed GABA_A_Rs with high zinc sensitivity indicating the prevalence of binary (α_x_β_x_) receptors over ternary (α_x_β_x_γ_x_) in these cells. The functionality of such receptors was demonstrated by Gunther et al. [41] in γ2 - subunit knockout mice. Taurine efficacy was comparable to GABA in zinc-sensitive cells, whereas taurine was less efficient than GABA in zinc-resistant cells, in keeping with the findings on recombinant γ-containing receptors expressed in *Xenopus* oocytes (Table 1), where taurine efficacy varied between 60–70% (α_1_β_3_γ_2,_ α_2_β_1_γ_1,_ α_2_β_1_γ_2_) and 80–90% (α_1_β_1_γ_2_, α_1_β_2_γ_2,_ α_2_β_3_γ_2_) of maximal GABA-responses. Note that in TMN neurons, which variably express 9 subunits of GABA_A_R [27], all aforementioned GABA_A_R types are likely occurring. The potency of taurine was not different between zinc-sensitive and zinc-resistant neurons, indicating that α_1_- and α_2_-containing GABA_A_R-populations, which show different changes in taurine potency upon co-assembly with the γ subunit (see above), may both contribute to the TMN pharmacology.

Receptors lacking a benzodiazepine (BZ) -binding site, such as α_1/2_β_1_, and α_1/2_β_1_δ, are better gated by taurine than by GABA (Table 1, Fig. 1). Our observation that taurine gating of β_3_-containing receptors with the same stoichiometry was weaker compared to β_1_-containing receptors could be explained by the presence of a low - affinity binding site for BZ at β_2/3_ but not at β_1_ receptors [42]. The mutation γ_2F77I_ which abolished zolpidem - potentiation did not rescue taurine gating. In contrast, taurine efficacy significantly dropped in this mutation, resembling now the taurine efficacy at the equivalent γ_1_ - containing receptors, which are poorly potentiated by a variety of BZ - site ligands [39] and naturally carry isoleucine at the position 77. We conclude that steric intersubunit - interactions (see below), rather than the BZ - binding site per se, play a decisive role for taurine or GABA gating as well as for the modulatory action of BZ.

In line with the data obtained on recombinant receptors containing the mutant γ_2F77I_ subunit, taurine efficacy was reduced in zinc - resistant native neurons from KI (γ_2F77I_) mice from 72% to 54% of maximal GABA efficacy. This efficacy drop corresponds very well to the values obtained from recombinant α_2_β_1_γ_2_ receptors (70% WT vs. 50% in α_2_β_1_γ_2F77I_ ) and supports our previous conclusion that the α_2_β_1_γ_2_ receptor type plays a dominant role for the pharmacology of TMN neurons [27]; [28]. Thus the mutation γ_2F77I_ modified taurine - efficacy at the γ_2_ - containing GABA_A_Rs. The (patho)physiological conditions for the gating of these receptors by taurine are unknown. The normal extracellular concentration of taurine is >20 times below their activation threshold. The expression of the α_4_ and δ subunits increases in the hippocampus of γ_2F77I_ KI mice, indicating that δ - containing receptors might be up -regulated as compensatory response for the impaired taurine efficacy at γ_2_ - containing receptors. This may be a reason for the lack of behavioural abnormalities in these mice compared to WT littermates [29]; [34]. Jia et al. [11] reported that, at extrasynaptic receptors of the α_4_β_2_δ-type, taurine shows agonistic properties superior to GABA and controls the excitability of mouse ventrobasal thalamic neurons. Jia et al. found a big difference in taurine sensitivity between recombinant α_4_β_2_δ receptors (threshold concentration 300 µM, EC_50_ = 7.5 mM) and native extrasynaptic receptors of possibly the same subunit composition (threshold concentration 10 µM, potency is not determined). In our study taurine showed higher potency at neuronal receptors (EC_50_ = 13–19 mM) compared to the corresponding recombinant α_2_β_1_γ_2_-receptors (EC_50_ = 120 mM) with threshold concentrations just above 1 mM. This disparity may result from the absence of GABA_A_R - associated proteins or yet unknown intracellular modulators in recombinant systems [11]. The exceptionally high potency and efficacy of taurine at α_4_β_2_δ receptors reported by Jia et al [11] together with the partial agonism of taurine at α_6_β_2_δ receptors [16] support our observation that the type of α subunit influences taurine binding or the transduction to receptor gating. Neither α_1_β_x_δ nor α_2_β_x_δ receptors in our study showed higher sensitivities to GABA when compared to the corresponding α_x_β_x_ receptors. This is in line with previous studies [43]; [44] where incorporation of the δ subunit was verified by concatenation. We applied tracazolate [35] at the end of each experiment to confirm the presence of a δ subunit in functional receptors. All data presented here are obtained from oocytes with different modulation of ternary versus binary receptors in parallel experiments. We cannot rule out the possibility of a sub-population of α_x_β_x_ receptors along with α_x_β_x_δ, which we tried to prevent by injection of 10∶1:10 cRNA ratios. Furthermore, significantly different parameters derived from the agonist concentration - response relationship (Table 1) indicates a prevalence of α_x_β_x_δ receptor types. We compared taurine agonism between the restricted number of GABA_A_R types expressed in histaminergic neurons and used δ- containing receptors (which are not expressed in TMN) only for the comparison with chimeric receptors, composed of the γ_2_ subunit with a δ-motif (MTVFLH). Structural determinants for super- and partial- agonism of taurine at δ-containing receptors await further characterisation.

The differences between β_1_ and β_3_ subunits seen in our study may rely on a number of subunit - specific residues involved in the stabilisation of receptor assembly. Bracamontes and Steinbach [45] described a number of β_3_ - specific residues allowing the formation of functional homomultimeric receptors. Some of them are located near or within the assembly signal, e.g. tyrosine at the position 81 (see Fig. 5, present study). Others are at remote places and unlikely involved in gating or agonist binding; they may play a role in the stabilization of different receptor conformations. Steric intersubunit interactions in heteromeric β_1_-containing receptors may support the stable transition from the closed to the open state after taurine binding at the α_x_β_1_δ, α_x_β_1_γ_2(δ74–79)_ and α_x_β_1_ receptor, with taurine acting as a superagonist. In contrast, at β_3_ - containing receptors taurine acts as a partial agonist compared to the analogous receptor types.

Our study reveals the importance of the γ_2_ motif around phenylalanine 77 for the reduction of taurine efficacy at γ - containing receptors and shows that all three subunit types (α_,_β_,_γ) in the GABA_A_ receptor can influence taurine agonism. Recent studies showed that the partiality of ligand agonism is pre - determined by the earliest step of agonist binding [46]; [47]. According to models suggested by these studies, partial agonist-binding generates an unstable conformational change, leading to receptor-flipping between closed and opened states [46]. Thus, the difference between taurine - and GABA - gating of GABA_A_R shown in this study indicates either a different but overlapping location of their binding sites or different transduction mechanisms at different receptor types.

By revealing structural demands for high efficacy GABA_A_R gating by taurine our study has broad physiological implications. Low taurine plasma level correlates with prediabetic and diabetic states and taurine supplementation is able to rescue insufficient insulin secretion by pancreatic islets [48]. Our data predict that a glucose-dependent up-regulation of the GABA_A_R γ2-subunit in pancreatic islets can reduce taurine action [49] and increase the risk of diabetes. (Patho)physiological correlates of GABA_A_R expression in pancreas await to be determined. Taurine deficiency in the brain results in GABAergic disinhibition, which models pathophysiological conditions of hepatic encephalopathy [12]. Thus the disclosure of structural demands for high efficacy taurine gating of GABA_A_R provides the basis for future studies analysing the role of GABA_A_R in diabetes mellitus and hepatic encephalopathy.

### Conclusions

Our study provides new insight into molecular determinants of taurine gating at γ - subunit containing receptors. The mutation of phenylalanine to isoleucine at position 77 in the γ_2_ subunit decreases, whereas introduction of the δ subunit-motif (MTVFLH) increases the efficacy of GABA_A_R gating by taurine. We show, that β_1_ (but not β_3_)-containing receptors display a wide range of taurine efficacies: from superagonism at α_x_β_1_ or α_x_β_1_δ receptors to partial agonism at γ-containing receptors. These findings shed light on the modification of GABA_A_R under (patho)physiological conditions accompanying the loss of endogeneous taurine, such as diabetes mellitus or hepatic encephalopathy.

## Supporting Information

Figure S1 **Zinc sensitivity of recombinant GABA_A_ receptors. (A)** Binary (α_x_β_x_, in black) GABA_A_ receptors are inhibited by 1 µM zinc, whereas ternary α_x_β_x_γ_2_ (dark grey) receptors are insensitive to zinc. Delta-containing α_x_β_x_δ receptors (in white) do not differ in zinc-sensitivity from the corresponding α_x_β_x_ receptors. Note that the zinc sensitivity is increased for αβγ_2(δ74–79)_ (light grey) receptors compared to the α_x_β_x_γ_2F77I_ receptors. Values represent mean ± SEM, p values are indicated by asterisk. * <0.05, ** <0.01, n.s. = not significant. **(B)** Representative current traces from oocyte recordings. Application is marked by horizontal bars. Scale markers represent 0.1 µA vertically and 20 s horizontally.
(PDF)

Figure S2 **Tracazolate (10** **µM)-potentiation of GABA-evoked currents is different between ternary δ-containing and corresponding binary α_x_β_x_ GABA_A_R types if GABA at ∼EC_10_ (for the β_1_-) and at ∼EC_99_ (for the β_3_-containing receptors) is used. (A)** When GABA concentration around EC_10_ is used, tracazolate-potentiation of binary α_x_β_1_ (but not α_x_β_3_) receptors is significantly smaller compared to the ternary δ-containing receptors. **(B)** When the same experiments were done at saturating GABA concentrations (∼EC_99_) ternary α_x_β_3_δ-GABA_A_ receptors were potentiated to a larger extent than the corresponding binary receptors. Note no difference between β_1_-containing ternary and binary receptors in experiments with this GABA concentration. p values are indicated by asterisk. * <0.05, ** <0.01, *** <0.001, n.s. = not significant.
(PDF)

Figure S3 **Zolpidem potentiation of different GABA_A_ receptor types. (A)** Zolpidem modulation of chimeric α_2_β_3_γ_2(δ 74–79)_ GABA_A_Rs. Introduction of the δ 74–79 motif MTVFLH into the γ_2_ subunit resulted in loss of potentiation by zolpidem, compared to the WT shown in (B). **(B)** Comparison of zolpidem-potentiation between α_2_β_3_γ_2_, α_2_β_1_γ_2_, α_2_β_1_γ_1_ and α_2_β_3_γ_2F77I_ receptors. Note much larger bi-phasic potentiation by zolpidem at β_3_-containing receptors (in contrast to the β_1_-containing receptors) in accordance with involvement of the low - affinity binding site for BZ at β_3_ but not at β_1_ receptors (44). This site is most likely responsible for the potentiation of GABA – responses at “zolpidem-resistant” (γ_2F77I_-containing) receptors by 100 µM zolpidem. Data represent mean ± SEM of at least 4 individual oocytes.
(PDF)
