# Correction: Partial Agonism of Taurine at Gamma-Containing Native and Recombinant GABA_A_ Receptors

**DOI:** 10.1371/annotation/fddd2ff3-c991-4c2f-8b84-a27eb20fba91

**Published:** 2013-10-24

**Authors:** Olaf Kletke, Guenter Gisselmann, Andrea May, Hanns Hatt, Olga A. Sergeeva

The name of the fifth author was incorrectly given in the citation. The correct citation is: Kletke O, Gisselmann G, May A, Hatt H, Sergeeva OA (2013) Partial Agonism of Taurine at Gamma-Containing Native and Recombinant GABA_A_ Receptors. PLoS ONE 8(4): e61733. doi:10.1371/journal.pone.0061733.

In addition, the order of affiliations is incorrect. The correct order of affiliations is: 1, Department of Neurophysiology, Medical Faculty of Heinrich-Heine University, Düsseldorf, Germany; 2, Department of Cell Physiology of the Ruhr-University, Bochum, Germany. 

