# Supplementary material for: Partial Agonism of Taurine at Gamma-Containing Native and Recombinant GABAA Receptors
Source: PLoS One. 2013 Apr 30;8(4):e61733. doi: 10.1371/journal.pone.0061733 (PMC3640040; doi:10.1371/journal.pone.0061733)
Supplement: Figure S1 — Zinc sensitivity of recombinant GABAA receptors. (A) Binary (αxβx, in black) GABAA receptors are inhibited by 1 µM zinc, whereas ternary αxβxγ2 (dark grey) receptors are insensitive to zinc. Delta-containing αxβxδ receptors (in white) do not differ in zinc-sensitivity from the corresponding αxβx receptors. Note that the zinc sensitivity is increased for αβγ2(δ74–79) (light grey) receptors compared to the αxβxγ2F77I receptors. Values represent mean ± SEM, p values are indicated by asterisk. * <0.05, ** <0.01, n.s. = not significant. (B) Representative current traces from oocyte recordings. Application is marked by horizontal bars. Scale markers represent 0.1 µA vertically and 20 s horizontally. (PDF) [file pone.0061733.s001.pdf]

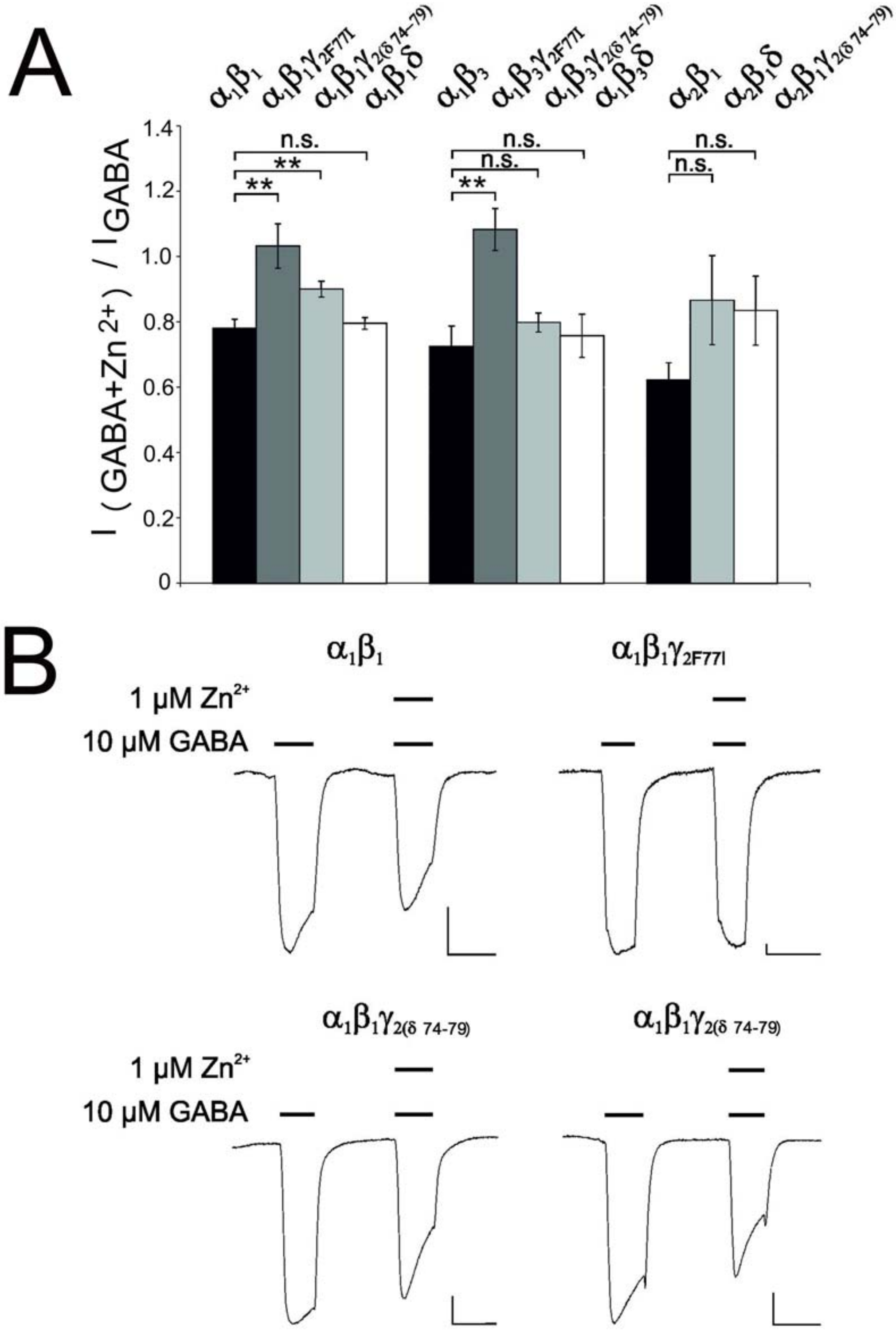

Supplementary Figure 1: Zinc sensitivity of recombinant $GABA_A$ receptors. (A) Binary ($\alpha_x\beta_x$, in black) $GABA_A$ receptors are inhibited by 1 µM zinc, whereas ternary $\alpha_x\beta_x\gamma_2$ (dark grey) receptors are insensitive to zinc. Delta-containing $\alpha_x\beta_x\delta$ receptors (in white) do not differ in zinc-sensitivity from the corresponding $\alpha_x\beta_x$ receptors. Note that the zinc sensitivity is increased for $\alpha\beta\gamma_{2(\delta 74-79)}$ (light grey) receptors compared to the $\alpha_x\beta_x\gamma_{2F77I}$ receptors. Values represent mean ± SEM, p values are indicated by asterisk. * < 0.05, ** < 0.01, n.s. = not significant. **(B)** Representative current traces from oocyte recordings. Application is marked by horizontal bars. Scale markers represent 0.1 µA vertically and 20 s horizontally.
