# Supplementary material for: Partial Agonism of Taurine at Gamma-Containing Native and Recombinant GABAA Receptors
Source: PLoS One. 2013 Apr 30;8(4):e61733. doi: 10.1371/journal.pone.0061733 (PMC3640040; doi:10.1371/journal.pone.0061733)
Supplement: Figure S2 — Tracazolate (10 µM)-potentiation of GABA-evoked currents is different between ternary δ-containing and corresponding binary αxβx GABAAR types if GABA at ∼EC10 (for the β1-) and at ∼EC99 (for the β3-containing receptors) is used. (A) When GABA concentration around EC10 is used, tracazolate-potentiation of binary αxβ1 (but not αxβ3) receptors is significantly smaller compared to the ternary δ-containing receptors. (B) When the same experiments were done at saturating GABA concentrations (∼EC99) ternary αxβ3δ-GABAA receptors were potentiated to a larger extent than the corresponding binary receptors. Note no difference between β1-containing ternary and binary receptors in experiments with this GABA concentration. p values are indicated by asterisk. * <0.05, ** <0.01, *** <0.001, n.s. = not significant. (PDF) [file pone.0061733.s002.pdf]

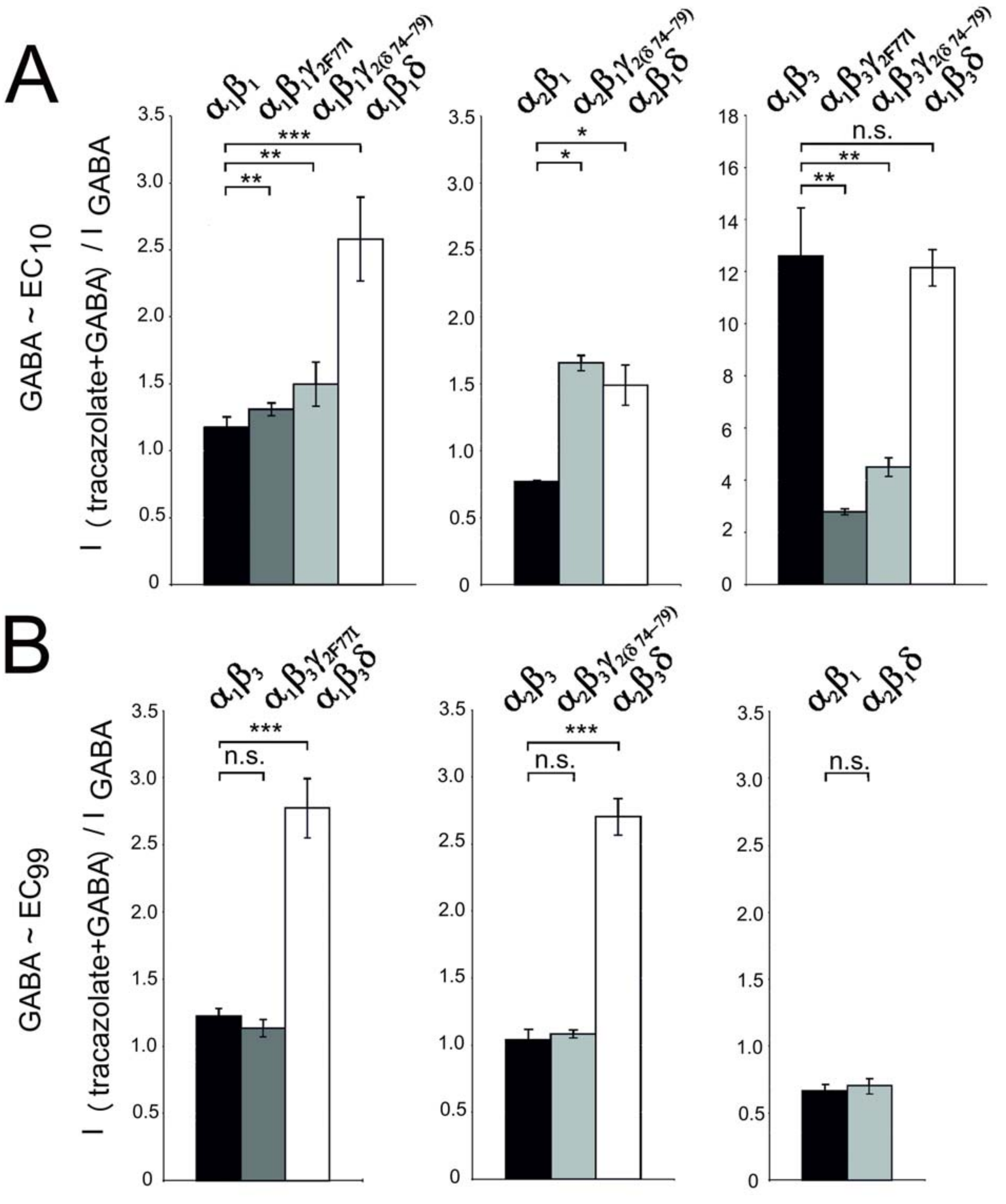

Supplementary Figure 2: Tracazolate (10 µM)-potentiation of GABA-evoked currents is different between ternary δ-containing and corresponding binary $\alpha_x\beta_x$ $GABA_AR$ types if GABA at ~EC_{10} (for the β_1-) and at ~EC_{99} (for the β_3-containing receptors) is used. (A) When GABA concentration around EC_{10} is used, tracazolate-potentiation of binary $\alpha_x\beta_1$ (but not $\alpha_x\beta_3$) receptors is significantly smaller compared to the ternary δ-containing receptors. **(B)** When the same experiments were done at saturating GABA concentrations (~EC_{99}) ternary $\alpha_x\beta_3\delta$-$GABA_A$ receptors were potentiated to a larger extent than the corresponding binary receptors. Note no difference between β_1-containing ternary and binary receptors in experiments with this GABA concentration. p values are indicated by asterisk. * < 0.05, ** < 0.01, *** < 0.001, n.s. = not significant
