# Supplementary material for: Partial Agonism of Taurine at Gamma-Containing Native and Recombinant GABAA Receptors
Source: PLoS One. 2013 Apr 30;8(4):e61733. doi: 10.1371/journal.pone.0061733 (PMC3640040; doi:10.1371/journal.pone.0061733)
Supplement: Figure S3 — Zolpidem potentiation of different GABAA receptor types. (A) Zolpidem modulation of chimeric α2β3γ2(δ 74–79) GABAARs. Introduction of the δ 74–79 motif MTVFLH into the γ2 subunit resulted in loss of potentiation by zolpidem, compared to the WT shown in (B). (B) Comparison of zolpidem-potentiation between α2β3γ2, α2β1γ2, α2β1γ1 and α2β3γ2F77I receptors. Note much larger bi-phasic potentiation by zolpidem at β3-containing receptors (in contrast to the β1-containing receptors) in accordance with involvement of the low - affinity binding site for BZ at β3 but not at β1 receptors (44). This site is most likely responsible for the potentiation of GABA – responses at “zolpidem-resistant” (γ2F77I-containing) receptors by 100 µM zolpidem. Data represent mean ± SEM of at least 4 individual oocytes. (PDF) [file pone.0061733.s003.pdf]

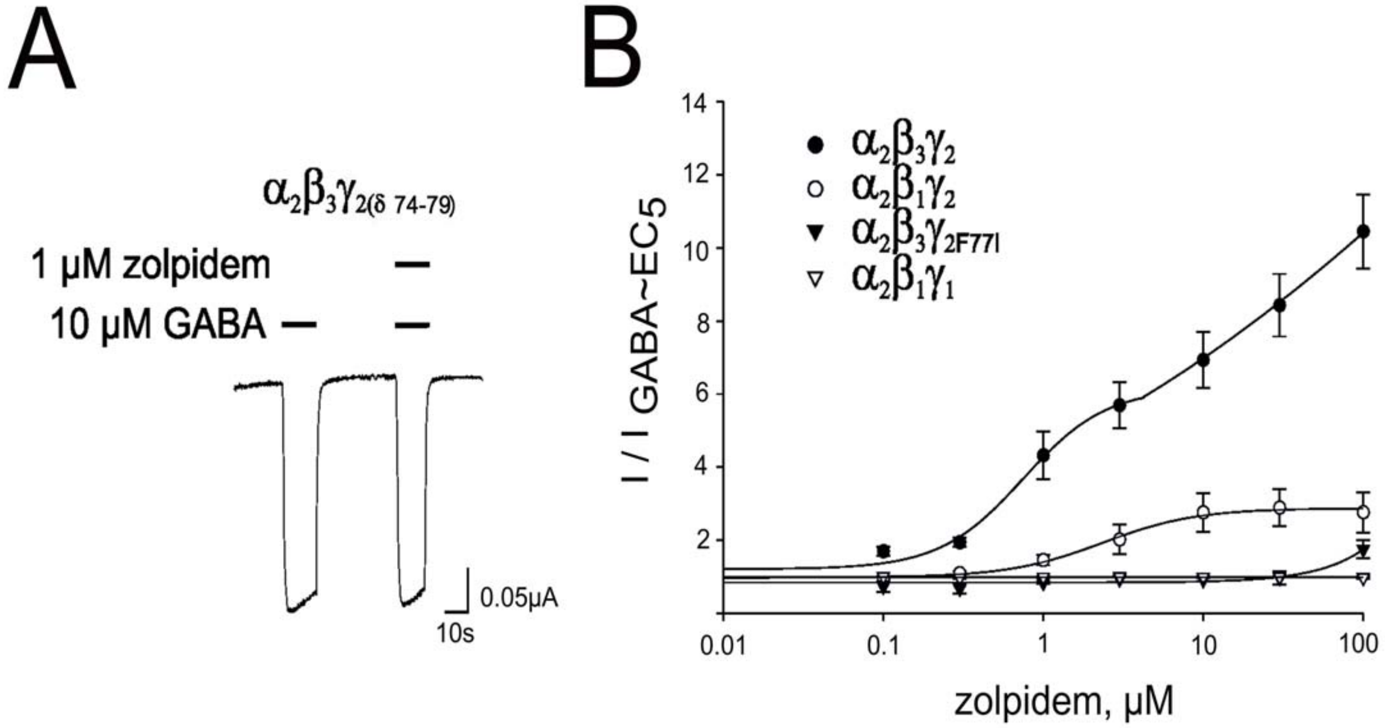

Supplementary Figure 3: Zolpidem potentiation of different $GABA_A$ receptor types. (A) Zolpidem modulation of chimeric $\alpha_2\beta_3\gamma_{2(\delta\ 74-79)}$ $GABA_A$Rs. Introduction of the δ 74-79 motive MTVFLH into the γ_2 subunit resulted in loss of potentiation by zolpidem, compared to the WT shown in (B). **(B)** Comparison of zolpidem-potentiation between $\alpha_2\beta_3\gamma_2$, $\alpha_2\beta_1\gamma_2$, $\alpha_2\beta_1\gamma_1$ and $\alpha_2\beta_3\gamma_{2F77I}$ receptors. Note much larger bi-phasic potentiation by zolpidem at β_3-containing receptors (in contrast to the β_1-containing receptors) in accordance with involvement of the propofol-binding site (β_{3N265}) into low-affinity benzodiazepine-modulation described previously (41). "Zolpidem-resistant" mutant γ_{2F77I}-containing receptors are potentiated by zolpidem starting with 100 μM. Data represent mean ± SEM of at least 4 individual oocytes.
